# Unveiling Fresh-Cut Lettuce Processing in Argentine Industries: Evaluating *Salmonella* Levels Using Predictive Microbiology Models

**DOI:** 10.3390/foods12213999

**Published:** 2023-11-01

**Authors:** Sofia Griselda Cuggino, Arícia Possas, Guiomar Denisse Posada-Izquierdo, Martin Gustavo Theumer, Fernando Pérez-Rodríguez

**Affiliations:** 1 Departamento de Fundamentación Biológica, Facultad de Ciencias Agropecuarias, Universidad Nacional de Córdoba, Córdoba X5000, Argentina; sofiacuggino@gmail.com; 2 Department of Food Science and Technology, UIC Zoonosis y Enfermedades Emergentes ENZOEM, CeiA3, Universidad de Córdoba, 14014 Córdoba, Spain; bt2poizg@uco.es (G.D.P.-I.); b42perof@uco.es (F.P.-R.); 3 Departamento de Bioquímica Clínica, Facultad de Ciencias Químicas, Universidad Nacional de Córdoba, Córdoba X5000, Argentina; mgtheumer@unc.edu.ar; 4 Consejo Nacional de Investigaciones Científicas y Técnicas (CONICET), Centro de Investigaciones en Bioquímica Clínica e Inmunología (CIBICI), Córdoba X5000, Argentina

**Keywords:** predictive microbiology, disinfection, food safety, cross-contamination, foodborne pathogens

## Abstract

A survey was performed to gather information on the processing steps, conditions, and practices employed by industries processing ready-to-eat (RTE) leafy vegetables in Argentina. A total of seven industries participated in the survey. A cluster analysis of the data obtained was performed to identify homogeneous groups among the participating industries. The data collected were used as inputs of two predictive microbiology models to estimate *Salmonella* concentrations after chlorine washing, during storage and distribution of final products, and to rank the different practices according to the final estimated *Salmonella* levels. Six different clusters were identified by evaluating the parameters, methods, and controls applied in each processing step, evidencing a great variability among industries. The disinfectant agent applied by all participating industries was sodium hypochlorite, though concentrations and application times differed among industries from 50 to 200 ppm for 30 to 110 s. Simulations using predictive models indicated that the reductions in *Salmonella* in RTE leafy vegetables would vary in the range of 1.70–2.95 log CFU/g during chlorine-washing depending on chlorine concentrations applied, washing times, and vegetable cutting size, which varied from 9 to 16 cm^2^ among industries. Moreover, *Salmonella* would be able to grow in RTE leafy vegetables during storage and distribution, achieving levels of up to 2 log CFU/g, considering the storage and transportation temperatures and times reported by the industries, which vary from 4 to 14 °C and from 18 to 30 h. These results could be used to prioritize risk-based sampling programs by Food Official Control or determine more adequate process parameters to mitigate *Salmonella* in RTE leafy vegetables. Additionally, the information gathered in this study is useful for microbiological risk assessments.

## 1. Introduction

The World Health Organization recommends the consumption of at least 400 g of fresh produce per day, while the Dietary Guidelines for the Argentine Population recommend the daily consumption of five servings of fruits and vegetables in a variety of types and colors [1,2]. However, it has been reported that only 6% of the Argentine population follows this recommendation [3]. Many factors contribute to consumers not incorporating fruits and vegetables into their diets, including socioeconomic status, availability of and accessibility to quality products, educational level, and, in some cases, the time required to prepare these foods [4,5]. The horticultural sector in Argentina is committed and challenged to optimize its production to promote an increase in fresh produce consumption, not only in terms of quantity and diversity but also in terms of quality [5,6,7,8].

While most vegetables are marketed as commodities or in bulk, another part is used for canning and dehydration. However, the production of fresh and ready-to-eat (RTE) products is still an emerging segment in Argentina, with little development in the agro-industrial sector [9,10]. These products require industrial processing that involves steps including selection and classification, cutting, washing-disinfection, centrifugations, and packaging. They are marketed as products for direct consumption in homes, restaurants, hotels, food chains, and catering services, among others [11]. In countries such as Spain, the production of fresh and RTE vegetables has been widely consolidated, offering the consumer healthy, fresh, and easy-to-consume or prepared foods, with high organoleptic quality [9,12,13,14]. 

Fresh-cut vegetables are consumed raw, without being subjected to any additional disinfection steps, which can completely eliminate any remaining pathogen contamination. Therefore, these products have been frequently implicated in foodborne outbreaks primarily related to contamination with pathogens such as *Escherichia coli* O157:H7 and *Salmonella* spp. [15,16,17,18]. According to Callejón et al. [15], *Salmonella* is a key pathogen responsible for foodborne illness outbreaks linked to fresh produce. Studies in South America have also reported the presence of *Salmonella* spp. in minimally processed vegetables [19,20,21,22].

The production chain of RTE vegetables is complex and encompasses several critical steps in which food safety may be compromised from a microbiological point of view [23,24]. Hence, a systematic approach, including all aspects of the production chain from “farm to table” is required, starting with the quality of the raw material, the effectiveness of washing, disinfection, and cross-contamination control through production, packaging, transport, and storage [25,26,27,28,29]. Additionally, several researchers have indicated that washing and sanitizing treatments constitute one of the most critical steps impacting the product quality, safety, and shelf-life [30,31,32,33]. Also, disinfection efficacy is affected by many factors such as the disinfectant agent, dosage, residual concentration, contact time, cut size, temperature, pH, and wash water/surface ratio where the sanitizer is applied [34,35,36,37].

The application of predictive microbiology models allows for estimation of the responses of microorganisms under specific environmental conditions along the food production chain, storage, and distribution [38,39,40]. Therefore, appropriate predictive models can be used to anticipate the growth, survival, and inactivation of microorganisms during the production–distribution chains of RTE vegetables, in order to evaluate the effect of different factors associated with each processing step (e.g., temperatures, times). In this sense, predictions can be useful for testing the efficacy of control measures or modifications of process parameters to prevent the exposure of consumers to microbial hazards.

A more specific knowledge of the practices and conditions employed by industries processing RTE vegetables can help to understand, prevent, and reduce contamination with pathogens throughout the production chain and ensure their microbiological safety. Therefore, the aims of this study were: (i) to collect information on the practices, parameters, and methods applied by RTE vegetable processing industries from different provinces of Argentina; and (ii) to use predictive models to estimate the impact of the washing and disinfection process with chlorine on the concentration of *Salmonella* in RTE vegetables, and evaluate whether storage and distribution practices applied in the industries affect pathogen survival or growth in the final product.

## 2. Materials and Methods

### 2.1. Participating Industries and Survey

Ten major RTE vegetables industries (processing plants, companies) located in different regions of Argentina were contacted for this study. First, contact was made through the e-mails published on their websites, and subsequent communications were established with the quality managers of each company. A questionnaire regarding the processing steps and parameters of RTE leafy vegetables production was elaborated, considering the most important factors affecting microbial fate in these types of products (e.g., temperature, time, sanitizer concentration ranges). The final version of the survey consisted of 28 questions with multiple answers, which were subdivided into six sections: (1) Description of industry and the processing steps carried out; (2 and 3) Data on the washing step and disinfection of RTE-leafy greens; (4) Details on the packaging conditions; (5) Information on post processing in-plant storage; and (6) Data on transport and distribution of the RTE leafy-green vegetables. The questionnaire was emailed to the quality managers of ten Argentine industries via GoogleForms^®^ (Google LLC, Mountain View, CA, USA) in March 2021.

### 2.2. Data Analysis

The R software (version 4.0.0) was used for graphical representation of the data derived from the questionnaire responses [41]. A cluster analysis was carried out using the InfoStat software, version 2017 (Grupo InfoStat, Córdoba, Argentina) [42], to identify the similar patterns among the surveyed industries considering the processing steps and conditions adopted by them.

### 2.3. Simulation Scenarios Using Predictive Models in the Processing and Conditioning Stages

Predictive models were used to quantitatively evaluate the impact of the processing steps, conditions, and practices adopted by the surveyed industries on levels of *Salmonella* in RTE leafy green vegetables. Thus, two models were selected for predicting the effect of distinct processing steps. 

First, the polynomial model by Cuggino et al. [36] was used to evaluate the disinfection efficacy of chlorine washing of fresh-cut lettuce, assuming products were contaminated with *Salmonella*. This model was developed based on the Response Surface Methodology and describes the *Salmonella* Thompson inactivation during chlorine washing as a function of four independent variables: free chlorine concentration (FCC, 0–200 ppm), contact time (30–110 s), cutting size (9–21 cm^2^), and benzyl isothiocyanate concentration (BITC, 0–80 ppm). It is important to highlight that the data to develop this model were obtained using model water at 4 °C and a water:lettuce ratio of 8.5 L/kg; therefore, these parameters were considered when evaluating the scenarios. 

Second, to evaluate the growth potential of *Salmonella* in the post-processing steps of fresh-cut lettuce, the secondary model by Cuggino et al. [43] was coupled with the primary model by Baranyi and Roberts [44]. Briefly, to develop this secondary model, *Salmonella* Thompson growth data over time were obtained in artificially contaminated fresh-cut lettuce at different storage temperatures (9–18 °C). The *Salmonella* growth parameters (i.e., growth rates, maximum population density) were estimated by fitting the Baranyi and Roberts model to the growth data. Then, the secondary model by Ratkowsky et al. [45] was used to relate *Salmonella* growth rates to the environmental temperature.

The abovementioned predictive models were implemented in the software MicroHibro (www.microhibro.com) for simulations. Once implemented into the software database, models become available in a user-friendly interface, in which users must define the conditions of predictions (e.g., initial level of contamination, chlorine concentration, environmental temperature) and the software returns *Salmonella* concentrations and estimated kinetic parameters. For simulations using the predictive models, the initial level of *Salmonella* contamination lettuce was set to be 3 log CFU/g, that is, the highest level that was reported in this type of product [36,46]. The parameters used as inputs for the predictive models to evaluate the different scenarios represented by each industry were the following: the average concentration of the disinfectant (ppm) applied during washing, the average disinfection time, and the cut size of the pieces of leafy vegetables (cm^2^), which varied among companies. The BITC was set to zero since none of the industries apply it in the disinfection process. The highest storage and distribution temperatures (°C) reported by the industries were used in the simulations. Therefore, worst-case scenarios in the disinfection and storage stages were used for the simulations using the predictive models. 

## 3. Results and Discussion

### 3.1. Description of the Participating Industries and the Processing Stages of Ready-to-Eat Leafy Vegetables

Seven out of the ten contacted industries answered the survey. They were identified with the letters A to G. Three of them are located in the province of Córdoba; two are located in Buenos Aires; one, in Mendoza; and one in Santa Fe. All of them are small enterprises with a maximum of 25 employees.

Two main stages were differentiated in all the companies: processing and conditioning. The processing stage included common processing steps, such as selection and classification of raw material, cutting process, washing-disinfection and centrifugation/dewatering; all of them being necessary for the transformation of vegetables into RTE vegetables [47]. Moreover, the conditioning stage included packaging, storage, and transportation of final products. The process stages informed by industries are those essential for obtaining vegetables ready for consumption, and have been previously reported [47,48,49].

**Figure 1:**
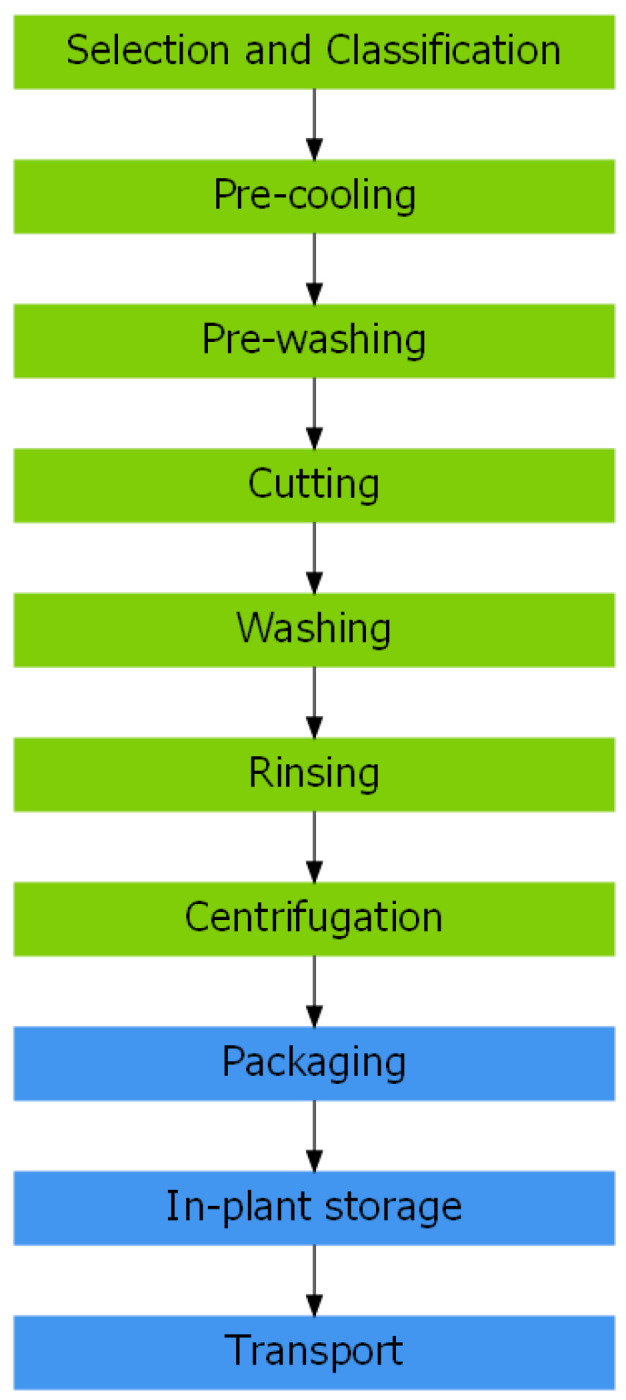
General process flow diagram of the elaboration of RTE leafy vegetables in Argentina. Processing (green boxes) and conditioning (blue boxes) stages.

A flow diagram showing the general process for RTE leafy vegetable production in Argentina was developed, based on the information provided by the seven participating industries (Figure 1). Despite the similarities in the processing and conditioning stages among industries, it should be noted that in some of them such as C, D, E, and F, the processing steps are totally automated, while in others, like A, B and G, some stages are manual.

### 3.2. Assessment of the Processing and Conditioning Stages of RTE Leafy Vegetables in Argentine Industries

**Figure 2:**
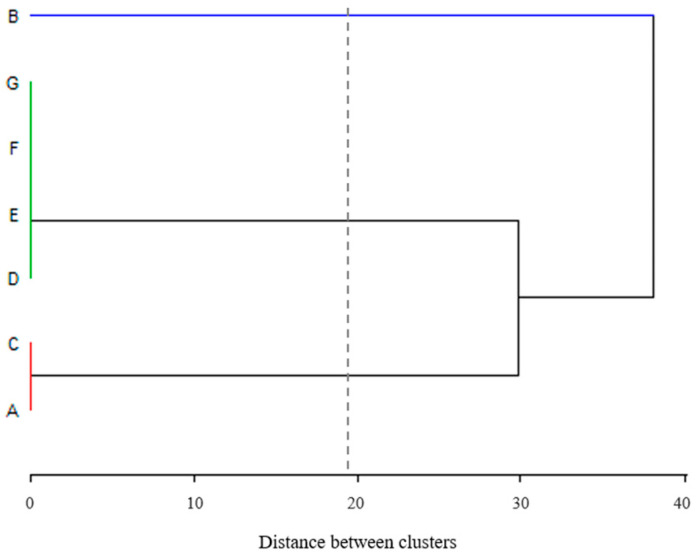
Cluster analysis of the processing and conditioning steps of ready-to-eat leafy vegetables performed by the seven participating industries (A–G).

To identify similarities or differences between the seven processing industries which participated in the survey, a cluster analysis was carried out using information about the processing and conditioning steps of RTE leafy vegetables available in the questionnaire responses. The results of the cluster analysis with regard to the steps carried out in the seven companies showed three major clusters, namely groups 1–3 (Figure 2). Group 1 (line blue), comprising industry B, showed a stark difference compared to the other groups, since it does not perform pre-washing, pre-cooling, or rinsing stages during the processing of leafy greens. Group 2 (line green), comprising industries D, E, F, and G, reported all the same processing stages shown in Figure 1. Group 3 (line red), comprising companies A and C, differ from company B in that they perform a rinsing stage, and from Group 2 in that they do not perform the pre-washing and pre-cooling stages.

**Figure 3:**
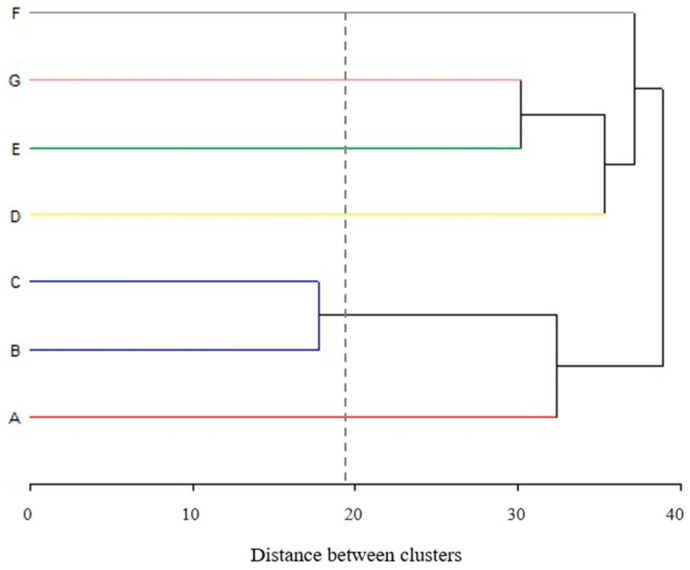
Cluster analysis of the parameters and methods used for the processing stages of ready-to-eat leafy vegetables in seven Argentine industries (A–G).

Furthermore, a cluster analysis was conducted, encompassing all parameters (e.g., temperature, times), methods, and controls employed at each stage. This yielded six distinct clusters (six different color lines), indicating significant variability among the practices and methods utilized by the various industries which participated in the survey (Figure 3).

The similarities and differences found in the processing and conditioning stages are detailed below.

#### 3.2.1. Processing Stage: Selection and Classification, Cutting, Disinfection, and Centrifugation Process

When analyzing in detail the common steps performed by the seven industries, similarities and differences were observed in some specific parameters and in the processing operations. For example, all participating companies select and classify their leafy greens, removing outer damaged leaves before processing. Although all seven industries perform the cutting operation, in companies A, B, C, D, E, and F lettuce is cut prior to disinfection, while in company G, lettuce is first disinfected and then cut. Concerning the vegetable cut sizes, they varied from 9 to 18 cm^2^, as shown in Table 1.

Another common step among all surveyed industries is disinfection; however, the parameters utilized vary among the different companies. For example, the temperature of the water used for the disinfection process was greater than or equal to 8 °C, and, in some cases, wash water at room temperature was used. As indicated by Gil et al. [30], leafy greens should cool quickly (less than 90 min) after harvest. This would slow down microbial growth in the case of contamination, and can be partially achieved by using wash water at low temperatures [32,50]. 

Also, all industries reported using sodium hypochlorite as a disinfectant for leafy vegetables during the washing operation, at concentrations ranging from 50 to 200 ppm. These results are in line with the fact that sodium hypochlorite remains the most widely utilized disinfectant in the fresh produce industry [32,51,52,53,54], due to its comparatively affordable price, ease of application, and extensive range of antimicrobial effectiveness [55,56]. 

**Figure 4:**
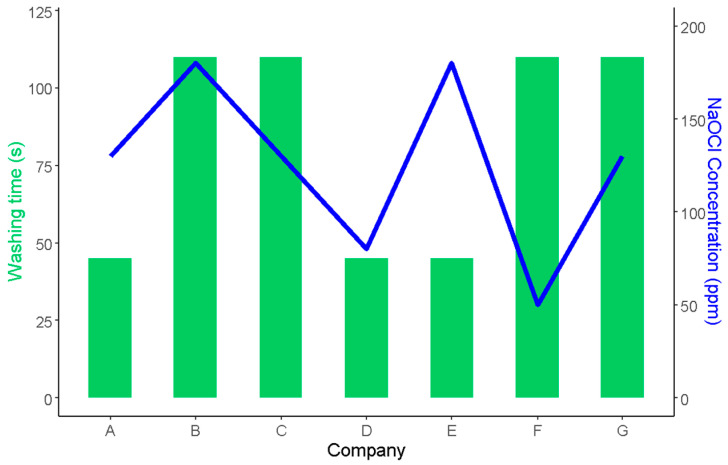
Sodium hypochlorite concentration and washing time used in seven ready-to-eat vegetable industries in Argentina (A–G). The bars represent the washing times, and the line represents the NaOCl concentration.

On the other hand, disinfection times also vary among companies, spanning from 30 to 110 s, as shown in Figure 4. This difference could be attributed to various factors unique to each industry’s processing needs and practices. Interestingly, despite this wide range of disinfection times, our analysis uncovered an unexpected outcome: no significant correlation existed between the concentration of chlorine and the duration of washing (Figure 4).

Addressing these differences in disinfection times and the disinfection concentrations used, optimization of the disinfection process takes on a crucial role. Ensuring the minimization of cross-contamination risks in the wash water while simultaneously curbing the formation of disinfection by-products (DBPs) is a pivotal consideration [57,58]. Consequently, maintaining a stringent and vigilant approach to monitoring the concentration of the disinfectant employed becomes imperative [31]. Also, it is noteworthy that the introduction of higher concentrations of chlorine, while potentially enhancing disinfection efficacy, also leads to heightened levels of DBPs in the wash water [31], requiring a thoughtful balance in their control as a key parameter within the disinfection process. 

According to the information provided by the quality managers of the industries, it is revealed that in some companies the pH of the wash water is not monitored (Table 1). It should be noted that there is much research showing that wash water pH is an essential parameter to control since the effective action of chlorine is highly dependent on pH, and its highest efficiency is at pH 7.5 [55,56]. It should also be highlighted that only company F adds citric acid as a pH regulator to adjust the value to 6.0–6.5. 

Concluding the analysis of survey results regarding the processing stages, following disinfection, all companies reported conducting a centrifugation process to eliminate the remaining wash water. By implementing this step, companies ensure the removal of excess moisture, which not only enhances the product’s shelf life but also contributes to preserving the desired sensory attributes of fresh produce [27,49].

#### 3.2.2. Conditioning Stage: Packaging, In-Plant Storage, and Transport

Packaging under hygienic conditions, carried out immediately after dewatering, plays an essential role in the microbiological protection of fresh-cut products [59,60]. The selection of the material, the conditions generated by the packaging, and the weight/volume relationship of the packaging are very important. For this reason, survey questions pertaining to packaging material and packaging conditions were included. Surprisingly, the findings demonstrate variations in the type of packaging material and conditions utilized, as specified by each industry (Table 1).

Industries A and B use plastic trays with film, without vacuum, while only companies C and G reported the use of plastic bags with modified atmosphere packaging (MAP), which is one of the most recommended methods for this type of products [34,56,61]. Modified atmosphere packaging has been introduced as an enhancement technology to extend shelf life of RTE vegetables, but it cannot always be implemented in small and medium-sized companies [62], which prioritize packaging solutions that require simple and economic technologies and materials.

Regarding in-plant storage, the finished products are stored for 12 h in all cases, except for industry A, where they are stored from 12 to 24 h. After storage, as reported by industry D, the products are transported for 12 h to the point of sale. In contrast, the other industries reported conducting transportation within 6 h. It is important to highlight that the RTE vegetables produced in industries A and B are transported at temperatures exceeding 8 °C. As described above, the temperature which RTE vegetables are exposed to has a direct impact on the sensory and microbiological quality of the products.

**Figure 5:**
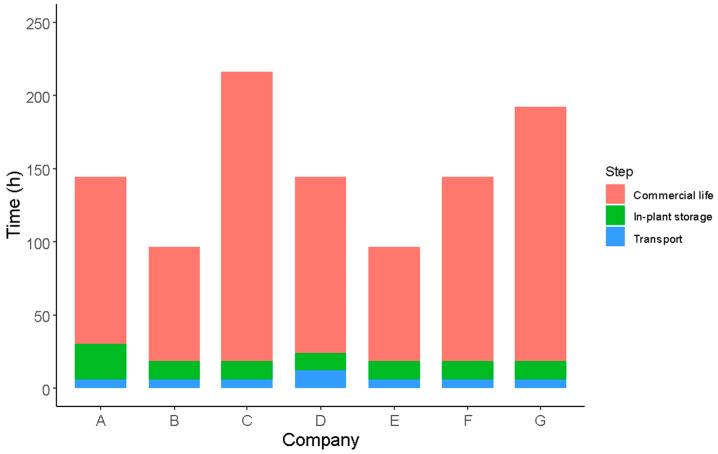
Relationship between packaging, storage and distribution time, and shelf life established for RTE leafy vegetables for industries A–G.

Regarding the declared shelf life of the products in the surveys, the finished products can be consumed while ensuring their quality and maintaining an appealing appearance for consumers in terms of color, hydration, crisp texture, and absence of discoloration, within a timeframe from 96 to 216 h (4 to 9 days) (Table 1). The reported findings do not show correlation between the type of packaging atmosphere used, storage and distribution times, and the declared shelf life (Table 1, Figure 5).

**Figure 6:**
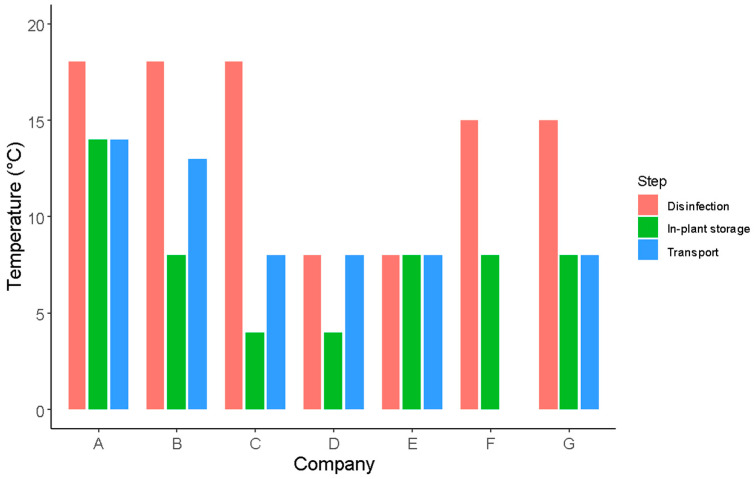
Exposure temperatures of leafy vegetables during processing (disinfection) and conditioning (in-plant storage and distribution) in industries A–G.

The exposure temperatures of leafy vegetables during processing and conditioning are depicted in Figure 6. As observed in the graph, there is significant variability in the temperature values reported by the industries.

Regarding the disinfection process temperature, none of the industries conduct this stage at temperatures below 8 °C. Concerning the storage stage of the final product in the industry, only industries C and D adhere to storage at temperatures below 4 °C, a temperature that inhibits microbial growth and reduces the respiration rate [13,63,64]. Particularly, company A indicated that products are disinfected, stored, and transported at temperatures exceeding 8 °C, posing a potential risk to the final product’s quality. This creates a favorable environment for microbial growth [34,61,62,65], which, in turn, impacts the organoleptic quality and the safety of the vegetables [34,48,61,64,66,67,68].

In addition, many studies have demonstrated that refrigeration is the most convenient and effective method to preserve organoleptic properties and extend the shelf life of fresh products [62,69,70,71]. However, to maintain a constant low temperature throughout the processing and distribution of RTE vegetables is a challenge and many small industries cannot economically afford to [48].

### 3.3. Evaluation of Salmonella Levels in Fresh-Cut Lettuce Using Predictive Models

#### 3.3.1. Disinfection Efficacy Using Chlorine Washing 

The model by Cuggino et al. [36] was applied to obtain estimations of *Salmonella* reductions in fresh-cut lettuce during chlorine washing. Seven different scenarios were considered for model simulations, each one representing the processing conditions of every participating industry (A–G). Table 2 shows the results obtained from the simulations of the predictive model.

As shown in Table 2, *Salmonella* reductions would range from 1.70 to 2.95 log CFU/g, depending on the conditions of the chlorine washing step. In other previous investigations, the effectiveness of chlorine in combination with disinfection time on the concentration of *Salmonella* resulted in similar reductions in fresh-cut produce [37,72]. Additionally, in accordance with the results of model simulations, in the study by Possas et al. [37] which modelled the *Salmonella* inactivation in fresh-cut lettuce, a maximum of 2.6 log-decrease in *Salmonella* levels were computed during chlorine washings at 50 to 150 mg/L for 0 to 2.5-min.

Specifically, the combination of parameters applied in industry G leads to a higher *Salmonella* reduction (i.e., 2.95 log CFU/g). On the contrary, the parameters set by company D show the lowest pathogen reduction (i.e., 1.7 log CFU/g). When comparing in detail the parameters and stages declared by industries A and G, it was observed that an increase in the disinfection time resulted in a greater reduction in *Salmonella*. Moreover, when comparing companies C and G, which apply the same chlorine concentration and washing times, it can be noted that the cut size can exert a slight effect in *Salmonella* levels, with a higher reduction when the size is bigger. This is interesting and evidences the need to consider vegetable surface area, in addition to other well-known intrinsic and extrinsic factors when estimating microbial growth, survival, or inactivation in fresh-cut lettuce. 

The provisions of Article 925 of the Food Code [73] in Argentina, and the Commission Regulation N° 2073/2005 [74] in Europe lay down that the absence (non-detection) of *Salmonella* must be ensured in 25 g of RTE vegetables. Thus, in a worst-case scenario of *Salmonella* contamination at 3 log CFU/g in unprocessed lettuce, none of the combinations of the parameters set by the seven industries would ensure the absence of the pathogen in 25 g. Although in company G the estimated *Salmonella* concentration after washing would be close to 0 (i.e., 0.05 log CFU/g), considering the uncertainty in the model predictions, absence of the pathogen cannot be assured. 

All steps of RTE vegetable processing play an important role in the quality of the final product, but the washing-disinfection step is key to achieving a reduction in microbial loads, while removing dirtiness and cell exudates [75]. However, if the chlorine concentrations are not well controlled, cross-contamination events during washing may occur and play a paramount role in the microbiological safety of the final products [25]. 

#### 3.3.2. *Salmonella* Growth Potential during In-Plant Storage and Distribution

The effects of in-plant storage and transport temperatures and times on *Salmonella* growth in fresh-cut lettuce following the chlorine washing step were evaluated by applying the model by Cuggino et al. [43]. The temperatures and storage and distribution times reported by the seven participating industries were used for model simulations (Table 3, Figure 6). This model has been developed with *Salmonella* growth data obtained from fresh-cut lettuce previously subjected to chlorine washing. The results of the model simulations are shown in Table 3. 

Since the growth model was developed in MAP fresh-cut lettuce, it was assumed for the simulations that all industries trade their products in this format. In addition, as the model was made for temperatures greater than or equal to 9 °C; a temperature of 9 °C was considered in model simulations for the companies that indicated carrying out storage and distribution at 8 °C, as this could be a possible scenario in the summertime.

Industries C and D indicated that the storage temperature of the products in the plant is 4 °C, in accordance with the recommended storage temperature for these products [76,77,78]. Therefore, a growth of *Salmonella* would not be expected during storage in companies C and D. However, since the disinfection treatment was not sufficient to fully eliminate *Salmonella* from their products, pathogen growth is expected during product distribution (Table 3). Considering the storage conditions (14 °C for 24 h), the highest increase in *Salmonella* levels would occur in products from company A (i.e., 0.86 CFU/g), compared to the other industries evaluated. On the other hand, the storage conditions of companies B, E, and F would also favor the growth of *Salmonella*.

After in-plant storage, RTE vegetables are distributed to retailers. Table 3 shows that, in all the scenarios evaluated, there would be an increase in the *Salmonella* concentration during the distribution step. In this sense, industry G should ensure no risk of cross-contamination with *Salmonella* during storage, since, after this step, the products are distributed at a higher temperature than recommended.

The application of the predictive microbiology models allowed for estimation of the responses of *Salmonella* in all the scenarios considered. It also highlights the importance of effectively combining an efficient disinfection step with the optimal control of storage and transport temperature of RTE vegetables to ensure the final quality and safety of the product. Overall, the relevance of applying predictive models implemented into user-friendly software lies in their ability to transfer knowledge to all food players involved in food safety, enabling a wide range of industries to make informed decisions, optimize processes, and enhance product quality and safety. More detail on the features and functionalities of MicroHibro can be found in Cubero-González et al. [79]. In addition to allowing a quantitative assessment of microbial responses in foods by means of microbiological models, MicroHibro includes a risk assessment module, a module for the design of sampling plans, and also a module which allows for food shelf-life assessment and estimation by means of quality models. Finally, an overall description of predictive microbiology models and risk assessments developed in recent years for fresh-produce can be found in Possas et al. [25]. 

## 4. Conclusions

The data collected in this study offer valuable insights into the diverse processes, methodologies, and practices employed throughout the production chain of RTE leafy vegetables in Argentina. Furthermore, in conjunction with previously published studies, this study plays a pivotal role in the identification and comprehensive evaluation of factors and process parameters that could significantly impact the safety of freshly processed products. The survey results highlight a significant disparity in practices and parameters employed by diverse industries involved in the production of RTE leafy vegetables in Argentina, confirming that knowledge and scientific development are often at odds with the industrial reality. The application of mathematical models enabled us to assess the impact of chlorine disinfection, as well as storage and distribution conditions, on the *Salmonella* levels in RTE lettuce, considering the conditions reported by fresh produce industries. Although chlorine disinfection is a crucial step for controlling pathogens in these products, model simulations confirmed that it is not enough to fully eliminate the pathogen since it can survive and then grow during subsequent storage and distribution stages. Despite the limited availability of public health data pertaining to diseases transmitted by RTE leafy vegetables, this research underscores the valuable role of surveys and predictive microbiology tools in guiding decision making and the evaluation of control measures within the fresh produce industry.

## Figures and Tables

**Table 1:** Parameters of the processing and conditioning stages of RTE leafy vegetables of seven Argentine industries (A–G).

Parameter	A	B	C	D	E	F	G
Cut size (cm^2^)	16	12	12	9	16	18	16
pH Control/pH	NA	NA	NA	6 to 8	NA	6 to 8	6 to 8
Packaging	Tray with film without vacuum	Tray with film without vacuum	Plastic bags MAP	PET containers with lid	Sealed plastic bags	Sealed plastic bags	Plastic bags MAP
Shelf life (h)	144	96	216	144	96	144	192

NA: not applied. MAP: modified atmosphere packaging.

**Table 2:** Reductions in *Salmonella* according to disinfection parameters set by the industries.

Industry	Chlorine Concentration (ppm)	Disinfection Time (s)	Cut Size (cm^2^)	Reductions in *Salmonella* (log CFU/g)
**A**	130	45	16	2.12
**B**	180	110	12	2.65
**C**	130	110	12	2.73
**D**	80	45	9	1.70
**E**	180	45	16	1.87
**F**	50	110	18	2.29
**G**	130	110	16	2.95

**Table 3:** Estimation of *Salmonella* growth in fresh-cut lettuce under different conditions of in-plant storage and transport.

Industries	Concentration of *Salmonella* after Disinfection ^1^ (CFU/g)	Storage Temperature (°C)	Storage Time (h)	Concentration of *Salmonella* after Storage (CFU/g)	Transport Temperature (°C)	Transportation and Distribution Time (h)	Concentration of *Salmonella* after Transport (CFU/g)
**A**	0.88	14	24	1.74	14	6	1.95
**B**	0.35	8	12	0.50	13	6	0.69
**C**	0.27	4	12	0.27	8	6	0.35
**D**	1.30	4	12	1.30	8	12	1.45
**E**	1.13	8	12	1.28	8	6	1.36
**F**	0.71	8	12	0.86	NR	6	-
**G**	0.05	8	12	0.20	8	6	0.28

^1^ Simulations conducted considering an initial *Salmonella* contamination of 3 CFU/g. NR: not reported.

## Data Availability

The data presented in this study are available on request from the corresponding author.
